# Applications of Functional Near-Infrared Spectroscopy (fNIRS) in Monitoring Treatment Response in Psychiatry: A Scoping Review

**DOI:** 10.3390/jcm14155197

**Published:** 2025-07-22

**Authors:** Ciprian-Ionuț Bǎcilǎ, Gabriela Mariana Marcu, Bogdan Ioan Vintilă, Claudia Elena Anghel, Andrei Lomnasan, Monica Cornea, Andreea Maria Grama

**Affiliations:** 1 Clinical Psychiatry Hospital “Dr. Gheorghe Preda”, 550082 Sibiu, Romania; ciprian.bacila@ulbsibiu.ro (C.-I.B.); claudia.anghel@ulbsibiu.ro (C.E.A.); andreilomnasan96@gmail.com (A.L.); moni.sipos@ymail.com (M.C.); andreeamaria.grama@ulbsibiu.ro (A.M.G.); 2 Faculty of Medicine, Lucian Blaga University of Sibiu, 550169 Sibiu, Romania; bogdan.vintila@ulbsibiu.ro; 3 Collective of Scientific Research in Neurosciences of the Clinical Psychiatry Hospital “Dr. Gheorghe Preda”, 550082 Sibiu, Romania; 4 Department of Psychology, Faculty of Social Sciences and Humanities, “Lucian Blaga” University of Sibiu, 550201 Sibiu, Romania; 5 County Clinical Emergency Hospital of Sibiu, 550245 Sibiu, Romania

**Keywords:** functional near-infrared spectroscopy, treatment response, treatment monitoring, psychiatric disorders, neuroimaging, brain activation scoping review

## Abstract

**Background/Objective**: Functional near-infrared spectroscopy (fNIRS) is a non-invasive neuroimaging technique with growing relevance in psychiatry. Its ability to measure cortical hemodynamics positions it as a potential tool for monitoring neurofunctional changes related to treatment. However, the specific features and level of consistency of its use in clinical psychiatric settings remain unclear. A scoping review was conducted under PRISMA-ScR guidelines to systematically map how fNIRS has been used in monitoring treatment response among individuals with psychiatric disorders. **Methods**: Forty-seven studies published between 2009 and 2025 were included based on predefined eligibility criteria. Data was extracted on publication trends, research design, sample characteristics, fNIRS paradigms, signal acquisition, preprocessing methods, and integration of clinical outcomes. Reported limitations and conflicts of interest were also analyzed. **Results**: The number of publications increased sharply after 2020, predominantly from Asia. Most studies used experimental designs, with 31.9% employing randomized controlled trials. Adults were the primary focus (93.6%), with verbal fluency tasks and DLPFC-targeted paradigms most common. Over half of the studies used high-density (>32-channel) systems. However, only 44.7% reported motion correction procedures, and 53.2% did not report activation direction. Clinical outcome linkage was explicitly stated in only 12.8% of studies. **Conclusions**: Despite growing clinical interest, with fNIRS showing promise as a non-invasive neuroimaging tool for monitoring psychiatric treatment response, the current evidence base is limited by methodological variability and inconsistent outcome integration. There is a rising need for the adoption of standardized protocols for both design and reporting. Future research should also include longitudinal studies and multimodal approaches to enhance validity and clinical relevance.

## 1. Introduction

Nowadays, medical practice is increasingly shifting towards individualized treatment, particularly in the field of mental health, where the variability of therapeutic responses necessitates an approach tailored to each patient [1]. This approach is supported by evidence-based medicine [2], which provides a rigorous framework for integrating the latest scientific data with the clinical experience of mental health professionals and the values and preferences of patients, thereby supporting the choice of a treatment approach tailored to their individual needs. In this context, the personalization of treatment must be accompanied by careful and continuous clinical monitoring [3], which allows for dynamic assessment of the efficacy and tolerability of the intervention. Monitoring is not a secondary step, but an essential component of the therapeutic process, as it enables the treatment to be adapted according to the evolution of symptoms and the patient’s individual characteristics [4].

Despite the advances in technology, contemporary psychiatry continues to lack objective, standardized tools, particularly tech-enabled systems, for monitoring brain activity during treatment, limiting the precision and reliability of clinical decision-making [5]. Although neuroimaging has guided psychiatric research for decades and promoted the development of brain biomarkers, progress has been limited by issues of reproducibility, low spatial and temporal resolution, heterogeneity of study populations, and the lack of standardized longitudinal approaches [6,7]. Thus, there is a growing need for the integration of complementary technologies, such as functional near-infrared spectroscopy (fNIRS), functional magnetic resonance imaging (fMRI), and quantitative electroencephalography (qEEG) in providing real-time neurophysiological data to support more accurate and individualized treatment monitoring [8,9]. The use of these methods in psychiatry is justified not only by the complexity of mental disorders and the limitations of conventional technologies but also by the increased prevalence of psychiatric disorders, which affect approximately one in eight people globally (WHO) [10]. In this landscape, fNIRS stands out as a noninvasive, portable neuroimaging method that allows continuous mapping of brain activity [11,12,13,14,15] by measuring regional cerebral blood volume (rCBV), expressed in terms of relative concentrations of oxyhemoglobin (HbO) and deoxyhemoglobin (HbR) at the scalp level [16,17,18].

The fNIRS technology uses two wavelengths in the near-infrared spectrum, and part of the emitted light is absorbed by hemoglobin during propagation from the source to the detector [19,20,21]. Based on the differences in absorption and applying the modified Beer–Lambert law [16,22], changes in HbO and HbR concentrations can be estimated. Neural activation causes blood vessels to dilate and regional blood flow to increase, exceeding local oxygen consumption, which is reflected by an increase in HbO [16]. The differences between the absorption coefficients of HbO and HbR allow the quantification of the relative concentrations in a brain region and provide information about local neuronal activity [23,24,25]. The detectors, also called optodes, capture light signals and, using certain mathematical models, estimate the values of the variations in HbO and HbR, which are correlated with the level of cortical activation [9,26]. The basic principle is a mechanism known as “neurovascular” coupling, whereby activation of a brain region is reflected by an increase in HbO concentration and a corresponding decrease in HbR levels [24,27,28].

An examination of the current landscape of neuroimaging techniques shows that fNIRS offers lower temporal resolution [29], but superior spatial resolution [9,30,31] when compared to EEG. fNIRS’s ability to sample brain signals at intervals of up to 0.1 s [19] places it above fMRI and PET in terms of temporal resolution [23,32]. In terms of spatial resolution, it remains inferior to that offered by fMRI [33], but fNIRS compensates for this with portability, low cost, and motion tolerance [20,34,35] being applicable in real time and under natural conditions, including in people performing certain activities [30,36,37,38]. In addition, fNIRS is the only functional neuroimaging technology that allows simultaneous measurement of variations in HbO, HbR, total hemoglobin (HbT), and cerebrospinal fluid (CSF), thereby facilitating the analysis of hemodynamic coupling stability [23,28]. Despite these advantages, some limitations should also be mentioned: shallow penetration depth, limited spatial resolution, and susceptibility to physiological artifacts (variations in blood pressure or blood flow in the scalp), which require caution in interpreting the data [14,33,39,40]. However, ongoing technological advances offer promising prospects for overcoming these limitations and expanding the clinical and experimental applicability of fNIRS [41].

In recent years, fNIRS has gained popularity in the fields of psychology and neuroscience [42], being widely used in the assessment of psychiatric disorders for diagnosis, differential diagnosis, and prediction of treatment efficacy [34]. By providing functional biomarkers of cortical activity with clinical relevance [43], it supports precision psychiatry approaches, which aim to tailor treatment to the individual patient profile through the integration of multimodal datasets and predictive algorithms, thereby enhancing the personalization of interventions and the efficient allocation of therapeutic resources [44,45,46]. In this regard, fNIRS has proven to be a promising and possibly effective method for monitoring treatment response in patients with psychiatric disorders, especially when other neuroimaging methods are limited.

Through this scoping review, we aim to (1) establish an operational definition of fNIRS use in clinical settings and (2) map the conceptual framework and application areas identified in the literature. Given the potential of fNIRS as a neuroimaging tool in psychiatry, our analysis focuses particularly on its application in monitoring therapeutic responses in patients with psychiatric disorders.

## 2. Materials and Methods

### 2.1. Transparency and Openness

This scoping review was conducted according to the Preferred Reporting Items for Systematic Reviews and Meta-Analyses Extension for Scoping Reviews (PRISMA-ScR) guidelines [47].

### 2.2. Scoping Review Research Questions

The primary research question guiding this review was: How has fNIRS been utilized to monitor treatment response in individuals with psychiatric disorders?

Based on this idea, we have developed the following questions:

(a) What methodological protocols and study designs have been used in fNIRS studies monitoring treatment response in individuals with psychiatric disorders?
(b) Which specific neurofunctional biomarkers derived from fNIRS (hemodynamic response, cortical activation, connectivity pattern) have been identified as relevant or predictive indicators of treatment efficacy in psychiatric disorders?
(c) What types of treatment (pharmacological, psychotherapeutic, neuromodulatory) were monitored?

Secondarily, the review also aims to identify trends in publications on the use of fNIRS for assessing treatment effects in psychiatric populations by identifying the psychiatric conditions studied (e.g., depression, schizophrenia, ADHD), and the correlation with clinical outcomes.

### 2.3. Search Strategy

The proposed scoping review has been conducted in accordance with the JBI methodology for scoping reviews [48,49]. We conducted bibliographic searches in four electronic databases: PubMed (MEDLINE), Embase, Web of Science, and Scopus. The search strategy was designed based on three key conceptual blocks combined with Boolean logic: (1) fNIRS, (2) psychiatric disorders, and (3) treatment monitoring/response. For each concept, we identified relevant Medical Subject Headings (MeSH) or Emtree (in Embase) terms and free-text keywords (terms in the title/abstract), which are provided in the Appendix A to ensure reproducibility. Each set of synonyms and terms was combined with OR, and the three concept blocks were then combined with AND (search syntax available in the Appendix A-Table A1).

### 2.4. Study Selection Procedure

**Figure 1:**
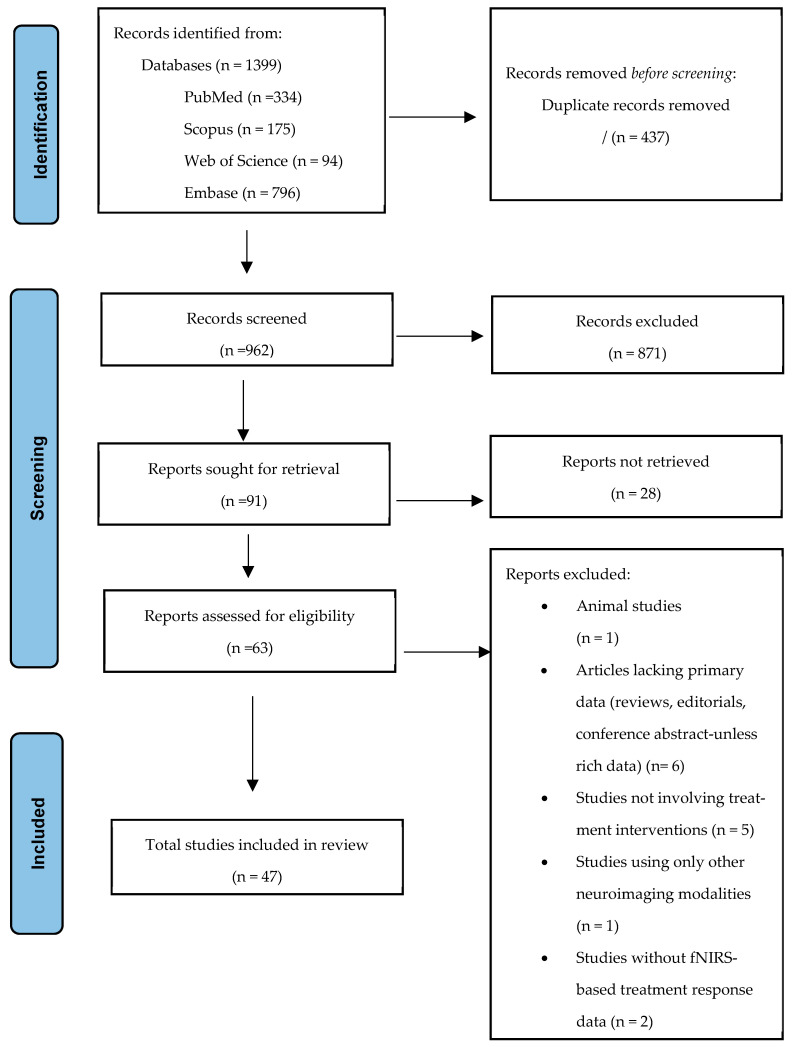
PRISMA flow diagram showing included studies at each stage of the inclusion/exclusion process.

A systematic literature search was conducted from March up until April 2025. The complete study selection procedure is illustrated in the PRISMA diagram (Figure 1). In the initial stage, the title and abstract were screened to identify articles eligible for full-text evaluation. All references retrieved from the database searches were imported into a reference management system and deduplicated. Database extraction and screening were undertaken by three authors using Rayyan (https://www.rayyan.ai, accessed on 20 March 2025), a web-based collaboration software platform, and duplicates were removed to facilitate review screening. Two independent reviewers, G.M.M. and A.M.G., screened titles and abstracts, followed by full-text reviews of eligible studies. Each study was labelled as “included,” “excluded,” or “unclear” by each reviewer. Reviewers did not have access to each other’s decisions at this stage to ensure independent assessments. Where required, conflicts were resolved by a third reviewer (C.I.B.) using a consensus method.

To be eligible for inclusion in this review, studies had to meet the following criteria: be peer-reviewed articles, include human participants diagnosed with psychiatric disorders (no age restrictions in clinical or research settings), use fNIRS to monitor treatment response, investigate any therapeutic intervention (including pharmacological, psychotherapeutic, or neuromodulatory); and utilize any study design (descriptive, observational, RCT, non-RCT, pilot) reporting original data on the use of fNIRS in this context.

Given the language skills of the evaluation team, only articles published in English were considered.

### 2.5. Data Extraction

The data extracted from each document included the following features:

(i) Publication characteristics, including study ID (author and year of publication), title, country of origin, and study design;

(ii) Sample characteristics, such as sample size, population type (children/adults), gender, age category of participants, and diagnosis;

(iii) Methodological features, including overall methodology, intervention or treatment type, and treatment duration;

(iv) fNIRS-related parameters, comprising the protocol and setup used, number of fNIRS channels, type of workload or task, brain regions or areas measured, motion correction techniques (e.g., moving average, PCA), signal filtering methods (e.g., bandpass), and signal transformation approaches (e.g., HbO/HbR computation); and

(v) Additional information, including declarations of conflicts of interest.

A structured summary of the included studies is presented in Appendix A (Table A2).

All these features have been analyzed and described in more detail below.

### 2.6. Data Analysis + Charting

The data were analyzed using quantitative (descriptive analyses) and qualitative (thematic and content analyses) methods. To outline a comprehensive framework on the current applicability of fNIRS in psychiatry, particularly in treatment response monitoring, the areas of interest of each publication were examined and coded into major thematic categories corresponding to general areas of application (such as mental health, clinical interventions, neuropsychiatric research, etc.). The nature of the fNIRS application (e.g., clinical, research, or experimental use) was also coded, and where relevant, the presence of critical discourse on its use was noted. The included studies were extracted into tables. Data charting was performed using an Excel (Version 16.97.25051114) spreadsheet, available by reasonable request. All the statistical analyses were performed in R [50].

### 2.7. Development of Concept

The use of fNIRS as a tool for monitoring treatment response involves applying this non-invasive neuroimaging method to objectively assess changes in brain activity associated with therapeutic interventions, thereby enabling the quantification of treatment efficacy and providing a potential functional biomarker in psychiatry.

### 2.8. Quality Appraisal

To ensure methodological rigor, we performed a formal quality appraisal of our scoping review using the 20-item Scoping Review Checklist [51] across five domains (study aim and research question; relevant studies; study selection; charting the data; collating, summarizing, and reporting results). Our review achieved a total score of 19 out of 20 (see Appendix A-Table A3 for the complete checklist and scoring details). In support of the criterion “The quality of papers was assessed”, we included a dedicated table detailing the presence or absence of quality-related issues across studies (see Appendix A Table A4).

## 3. Results

### 3.1. Descriptive Mapping of Studies

**Figure 2:**
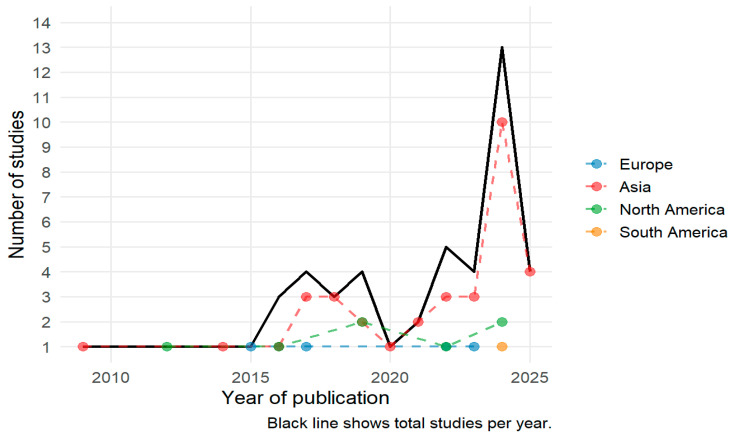
Annual distribution of fNIRS publications on monitoring treatment response in psychiatric populations.

A total of 47 studies met the inclusion criteria for this scoping review. These studies, published between 2009 and 2025, reflect a growing interest in the application of fNIRS for monitoring treatment response in psychiatric populations. Figure 2 presents the annual distribution of publications, illustrating temporal trends in the use of fNIRS as a neuroimaging tool within clinical psychiatry.

#### 3.1.1. fNIRS Study Publication Trends by Year and Origin

The use of fNIRS to assess treatment response in psychiatric populations has increased over the past decade, with a sharp rise in publications observed after 2020. This growth is driven primarily by studies conducted in Asia [16,32,34,37,42,43,44,45,46,52,53,54,55,56,57,58,59,60,61,62,63,64,65,66,67,68,69,70,71,72,73,74], which account for the majority of contributions in recent years. Europe [17,18,75,76,77], South America [35], and North America [78,79,80,81,82,83] also show modest but steady activity. This overall increasing trajectory suggests a progressive integration of fNIRS into research and clinical frameworks aimed at evaluating neurofunctional changes associated with psychiatric interventions.

#### 3.1.2. Research Designs and Sample Composition Across Included Studies

Among the 47 studies included, most adopted experimental methodologies, with 31.9% employing randomized controlled designs and 23.4% using non-randomized trials [16,42,55,58,61,73,78,81,83,84]. Observational (21.3%) [32,34,36,37,43,44,75,80,82,85], pilot (17.0%) [18,57,64,65,66,67,70,79], and descriptive/exploratory (6.4%) [56,59,68] designs were less frequent, highlighting a general trend toward hypothesis-driven clinical fNIRS research. Over half of the studies (68.08%) enrolled samples between 21–100 participants, while 8.51% exceeded 100 [32,34,36,72]. Small samples (≤20) [16,44,57,59,61,64,67,68,83] were observed in 19.14% of studies, and there were two studies [56,84] using single-subject designs.

**Figure 3:**
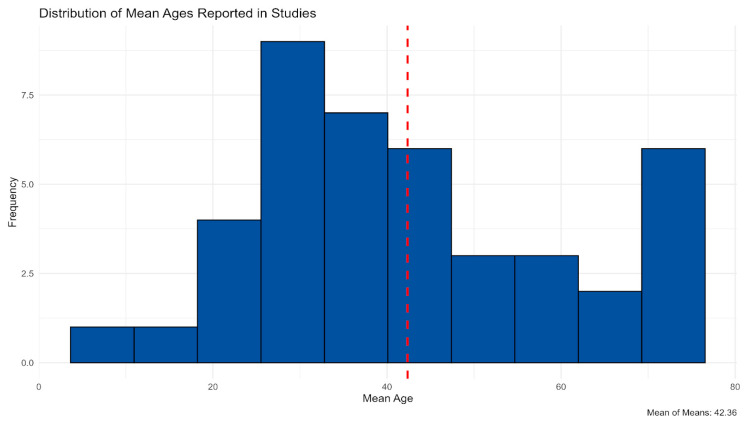
Age distribution across studies.

The majority of samples comprised adults (93.6%), with few studies targeting children (6.4%) [64,69,81]. Most studies included mixed-gender samples (85.1%), though a small subset investigated only male (10.6%) [34,53,56,62,65] or female (4.3%) [67,84] participants. Age distribution (Figure 3) skewed toward adults aged 18–64 (44.7%), with additional representation of older adults (12.8%) and mixed-age samples (25.5%). Very few studies focused exclusively on children or adolescents (4.3% each), and 4.3% did not report age [59,82].

#### 3.1.3. Study Characteristics: fNIRS Paradigms, Signal Acquisition, and Analytical Approaches

fNIRS was most commonly applied in task-based paradigms (63.8%), followed by resting-state designs (29.8%) [18,35,42,43,53,56,57,60,66,71,72,76,81,83] and mixed paradigms (6.4%) [32,45,78]. Over 80% of studies involved multiple-session interventions, with only 14.9% measuring neural change after a single session [17,18,35,63,67,68,83]. The most frequently used tasks were verbal fluency tests (29.8%), cognitive tasks (12.8%) [67,69,79,80,82,84], and emotional or autobiographical recall paradigms (10.6%) [16,59,62,77,85]. While 23.4% of studies used resting-state or no-task designs, a notable 14.9% employed combined tasks [17,43,45,60,66,68,74,78] integrating multiple cognitive or affective components.

Signal acquisition varied considerably, with more than half of studies (54.5%) using systems with >32 channels, supporting whole-brain or broad frontal-parietal coverage. A smaller number of studies used 17–32 (17.02%) or fewer channels (≤16: 31.9%), often targeting prefrontal regions. The dorsolateral prefrontal cortex (DLPFC) was the most frequently measured region (55.3%), followed by broader prefrontal cortex (PFC) coverage (23.4%). Only a minority targeted temporal regions (10.6%), and none focused solely on parietal or orbitofrontal areas.

Analysis pipelines were heterogeneous. Most studies (74.5%) explicitly described artifact removal procedures, typically employing motion correction or filtering. A smaller fraction (6.4%) [16,56,78] did not apply preprocessing, and 19.1% failed to report it [18,35,36,37,42,54,55,57,69]. The majority of studies (83.0%) used commercial fNIRS systems, while a few employed wearable or custom-built platforms. Analysis software varied, with 40.4% relying on manufacturer software, while others used open-source toolboxes like HOMER2 (27.7%) or general platforms such as MATLAB (17.0%). Only 2.1% of studies reported concurrent multimodal neuroimaging, typically EEG, with 70.2% using other types of auxiliary equipment (e.g., behavioral tasks or physiological monitors).

Characteristics of the included studies are summarized in Table 1, with no missing values for the variables reported.

### 3.2. fNIRS Applications by Psychiatric Disorder and Intervention Type

#### 3.2.1. Distribution of Studies Across Disorders and Interventions

**Figure 4:**
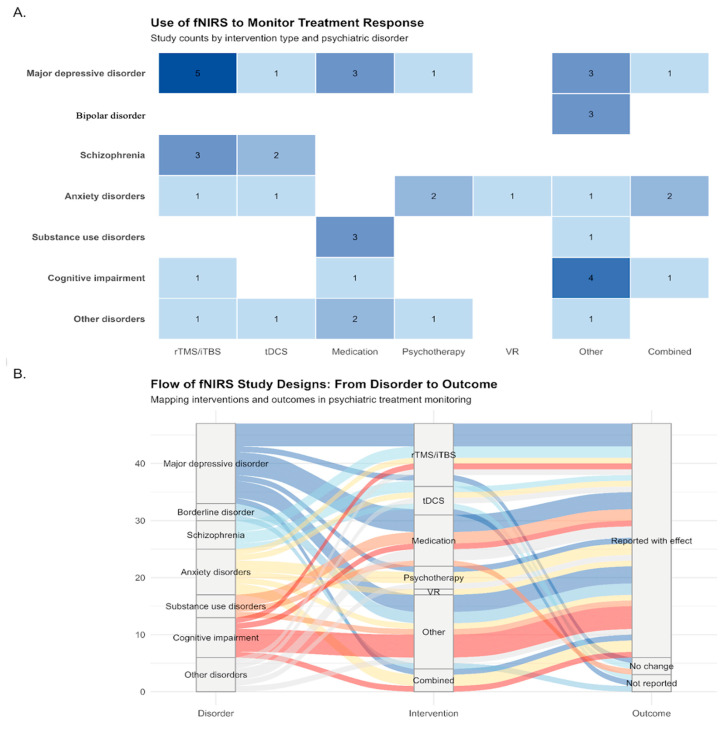
Use of fNIRS to Monitor Treatment Response Across Psychiatric Disorders. (**A**) Heatmap showing the number of studies employing fNIRS across different psychiatric diagnoses and intervention types. Most research has focused on MDD, schizophrenia, and anxiety disorders, particularly in conjunction with rTMS/iTBS and pharmacological interventions. (**B**) Sankey diagram illustrating the flow from diagnosis to intervention and reported clinical outcomes. The majority of studies report positive fNIRS-clinical correlations, though representation varies across disorders. Gaps in combined or VR-based therapies suggest potential avenues for future research.

A heatmap was generated to illustrate the number of studies employing fNIRS to monitor treatment response across different psychiatric diagnoses and intervention types (Figure 4A, upper panel). The most commonly studied conditions were MDD, schizophrenia, and anxiety disorders. In terms of interventions, repetitive transcranial magnetic stimulation (rTMS/iTBS) and pharmacological treatments were most frequently investigated. This distribution suggests that fNIRS is primarily utilized in well-established clinical populations and in conjunction with neuromodulatory or pharmacological approaches. Conversely, fNIRS remains underutilized in emerging or integrated treatment modalities, such as virtual reality (VR)-based or combined interventions, highlighting potential directions for future research.

#### 3.2.2. Mapping Disorder—Intervention—Outcome Relationships

To further explore how fNIRS has been applied across diagnostic categories, interventions, and clinical outcomes, a Sankey diagram was constructed (Figure 4B, bottom panel). The diagram illustrates how fNIRS has been applied across psychiatric disorders, interventions, and reported outcomes. The majority of studies focus on MDD, primarily using rTMS/iTBS and medication, and most report a positive link between neural changes and clinical improvement. Cognitive impairment and schizophrenia studies are more methodologically diverse but also tend toward positive outcomes. Disorders like anxiety, BD, and SUD are underrepresented, with less consistent outcome reporting.

#### 3.2.3. fNIRS-Clinical Outcome Associations Across Studies

**Figure 5:**
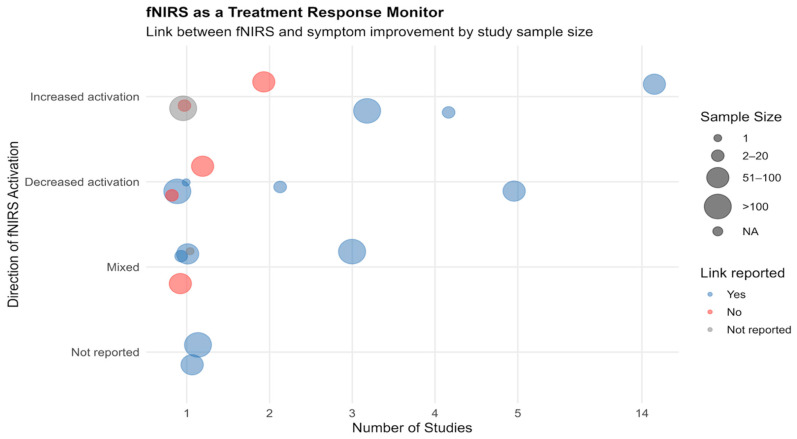
Bubble plot to illustrate fNIRS use to monitor treatment-related brain activation in relation to symptom improvement across psychiatric studies (N = 47). The x-axis represents the number of studies reporting each combination of activation pattern and clinical link, while the y-axis categorizes the direction of fNIRS activation observed post-treatment: increased, decreased, mixed, or not reported. Each bubble is sized according to sample size category, ranging from single-subject designs to studies with over 100 participants. Bubble color indicates whether a link between fNIRS signal change and clinical improvement was reported (Yes = blue, No = red, Not reported = grey).

Reporting of associations between fNIRS-measured brain activation and clinical symptom improvement was inconsistent across studies (Figure 5). Among the 47 studies, 39 studies (83.0%) explicitly reported a positive association between fNIRS activation and symptom improvement, while 2 (4.3%) [77,84] reported no link. The remaining six studies (12.8%) [18,66,67,76,83,85] did not state whether such a link was examined or found.

Direction of fNIRS activation also varied. Increased activation was the most frequently reported pattern (25.5%), followed by mixed patterns (17.0%) and decreased activation (4.3%). Most studies that did report a positive link (blue bubbles) used small to mid-sized samples (2–100 participants), with some clustering seen around increased or mixed activation patterns. A small number of studies (red bubbles) explicitly found no association, and these also included various activation directions. The limited number of studies clearly reporting both activation direction and clinical outcome reflects a common issue in the field: incomplete or absent reporting of either clinical outcomes, fNIRS signal direction, or both. Notably, 25 studies (53.2%) did not report the direction of neural activation, indicating a lack of standardized reporting in this domain.

### 3.3. Preprocessing Approaches in fNIRS Signal Analysis

#### 3.3.1. Motion Artifact Correction

Motion correction procedures were reported in 44.7% of the included studies (n = 21), while the remaining 55.3% (n = 26) did not apply or report any such correction. Among studies that implemented motion correction, the most frequently used methods involved MATLAB-based toolkits, particularly the HOMER2/HOMER3 preprocessing pipelines (n = 7). A smaller number employed principal component analysis (PCA)-based corrections (n = 2) and smoothing techniques using moving average filters (n = 2). Less commonly applied approaches included Temporal Derivative Distribution Repair (TDDR) (n = 2), coefficient-of-variation-based detection (n = 1), wavelet-based artifact removal (n = 1), and spline interpolation (n = 1). A few studies also relied on system-specific solutions, such as the ETG-4000 preprocessing pipeline (n = 1), or open-source alternatives like PoTATo Toolbox (n = 1). Three studies used other or unspecified methods.

#### 3.3.2. Signal Filtering

Filtering approaches varied notably across studies. Bandpass filtering, which applies both high- and low-frequency cutoffs, was the most common, used in 39.5% of studies (n = 15). Only 7.9% (n = 3) [36,59,80] used low-pass filtering alone, while 44.7% (n = 17) did not report their filtering strategy. An additional 7.9% (n = 3) [59,76,80] employed ambiguous or unconventional filtering methods that could not be clearly classified. Among studies that reported specific cutoff values, high-frequency cutoffs ranged between 0.01 and 0.5 Hz, and low-frequency cutoffs ranged from 0.005 to 0.2 Hz, consistent with standard recommendations for physiological noise reduction.

#### 3.3.3. Signal Transformation

A total of 48.9% of studies (n = 23) explicitly reported the use of the modified Beer–Lambert law (MBLL) to convert raw optical density signals into hemoglobin concentration changes. In contrast, 27.7% (n = 13) did not report the signal transformation method used [17,36,56,62,63,64,66,67,69,76,77,83], and 23.4% (n = 11) applied other or unclear transformation approaches, including unspecified or customized algorithms.

### 3.4. Reported Limitations and Conflict of Interest

Of all the studies included, 45.07% (n = 32) reported small sample size as a limitation, 16.90% reported having no control/placebo/sham/healthy group (n = 12), 14.08% reported technical limitations (e.g., resolution issues, not enough NIRS channels; n = 10), and 14.08% reported having a short/no follow-up or no long-term measurements (n = 10, 10.94%). Limited generalizability/high heterogeneity (n = 6, 8.45%) was also mentioned, and one study [77] reported blinding issues. The summary of limitations reported is presented in Table 2.

The majority of studies had an explicit conflict of interest section (n = 43, 91.48%), while 8.52%% (n = 4) did not report any such statement. Among those that did report, the vast majority (77.6%, n = 38) explicitly stated that no conflicts of interest were present. A smaller portion (8.16%, n = 4) reported potential commercial or financial conflicts, typically involving research funding, personal fees, or equity holdings by one or more authors.

## 4. Discussion

This is the first scoping review that presents an overview of the current literature on the use of fNIRS in monitoring treatment response in psychiatric disorders, highlighting the diversity of methodologies employed and identifying areas where research practices could be optimized.

The overall trajectory of the field points to a substantial increase—with a notable acceleration after 2020—in the use of fNIRS as a monitoring tool for treatment response in psychiatry. This upward trend suggests a gradual integration of fNIRS into clinical neuroimaging research, reflecting the growing interest in non-invasive neurofunctional biomarkers of treatment efficacy. However, the concentration of research in a limited number of countries might hinder generalizability across diverse healthcare systems and populations.

### 4.1. Study Designs, Samples and Clinical Focus

Most studies have adopted an experimental design, with nearly one third (31.9%) using randomized controlled trials (RCTs), demonstrating efforts to scientifically inform clinicians of fNIRS’s utility as a treatment monitoring tool.

Yet, a significant proportion relied on small or moderate sample sizes (<100 participants), reducing statistical power and highlighting the need for future studies with larger cohorts or multicenter approaches.

Nearly all studies focused on adult populations of both sexes, probably because their cooperation is generally easier to obtain. In contrast, children and adolescents were underrepresented. This sample profile limits the generalization of fNIRS treatment monitoring utility outside the adult population, highlighting the need to include children and adolescents in future research, with a particular take on the neurobiological development specific to these age groups.

The distribution of studies according to diagnosis reveals that fNIRS was most often applied to populations with MDD, followed by schizophrenia and anxiety disorders. This dominant focus reflects a preference for well-established psychiatric conditions while other conditions of presumably high interest (such as BD or substance abuse disorder) were not approached. The concentration of evidence for MDD and, to a lesser degree, schizophrenia may also reflect the unique suitability of fNIRS for detecting changes in frontal lobe function—a region centrally involved in both conditions. In everyday psychiatric practice, this means that fNIRS-based monitoring may be most immediately useful for clinicians treating patients with disturbances in cognitive, executive, or emotional regulation.

### 4.2. Experimental Paradigms and Neural Targets

Experimental approaches have primarily focused on task-based paradigms and multi-session interventions, with an emphasis on frontal activation through verbal-fluency tasks. The frequent use of the verbal fluency test (VFT) in nearly one-third of studies highlights its effectiveness in activating the prefrontal cortex, making it particularly useful in the neurofunctional investigation of depression [36,52,55,58,61] and dementia [27]. In contrast, resting-state or mixed designs have been less common despite their advantage to capture both spontaneous activity and reactivity to stimulation. Similarly, only a few studies incorporated multimodal neuroimaging to validate fNIRS findings. A few studies have employed simultaneous EEG, providing complementary information about electrical versus hemodynamic neural activity; however, such approaches remain rare. Multimodal integration has the potential to enhance understanding of treatment mechanisms (e.g., by correlating hemodynamic changes with electrophysiological markers of cortical excitability) [32,65]. Future research could expand in this direction, given the compatibility of fNIRS with EEG and the potential to obtain a more comprehensive neurophysiological picture in real time. The methodological preference for monomodal measurement reflects the fact that fNIRS was primarily used to track the gradual evolution of the brain’s response to treatment rather than as a tool for assessing immediate effects [79,83].

Most of the studies targeted the DLPFC as the primary region of interest, which aligns with the established role of the DLPFC in executive and affective regulation. This focus on the frontal lobe reflects the hypothesis that improvement in psychiatric symptoms is associated with functional changes in frontal networks, particularly through a reduction in the hypofrontality characteristic of pre-treatment depressive states and normalization of DLPFC activation after intervention (neurobiological + prefrontal brain function). On the other hand, only a few studies targeted other cortical areas, such as temporal regions, while other cortical areas were not included at all (parietal and orbitofrontal) limiting the exploration of disorder-specific patterns of activation involved in the therapeutic response [70], beyond the frontal cortex.

The technical characteristics of the fNIRS systems used differ significantly between studies, which may influence the cortical area covered and the granularity of the data obtained.

The processing and analysis of fNIRS data are not yet standardized, with considerable heterogeneity between studies in terms of analytical pipelines. Most authors have reported the application of preprocessing methods to remove artifacts and noise, the most common being motion corrections and signal filtering. However, some studies either did not apply motion corrections or did not report this, which is concerning given the susceptibility of the fNIRS signal to motion artifacts [34,66]. The lack of these details complicates the reproducibility and comparison of results between studies, which are essential for establishing the validity of any neuroimaging method.

The diversity of analysis software, ranging from commercial software to open-source platforms such as HOMER2 or MATLAB, reflects the lack of both analytical and reporting standardization. Some authors did not specify the method used at all, while others resorted to either algorithms integrated into the equipment software or customized approaches. This variability raises issues regarding the comparability of response amplitudes reported across studies, as without standardization in data conversion, differences in activation magnitude may partly reflect procedural differences rather than biological differences [86].

As fNIRS protocols remain heterogeneous, clinicians should approach current findings with caution, understanding that methodological discrepancies can impact their clinical applicability.

### 4.3. Clinical Outcome Integration and fNIRS Treatment Monitoring Potential

The most frequently investigated interventions actually corresponded to the mostly used therapeutic approaches in the main psychiatric conditions: transcranial magnetic stimulation (rTMS/iTBS) and pharmacological treatments. This suggests that fNIRS was primarily employed in established clinical contexts and in conjunction with biological interventions (neuromodulatory or pharmacological) approaches, where measurable changes in neural activation are anticipated. On the other hand, fNIRS remains underutilized in newer or integrated therapeutic modalities. Interventions such as virtual reality (VR) therapy, biofeedback/neurofeedback training, or complex combinations have rarely been investigated with fNIRS. This highlights opportunities for expanding future research to verify the utility of fNIRS in these less explored contexts [46]. For example, almost no studies in the sample examined cognitive rehabilitation interventions or computerized training, which could be a promising future direction, given that fNIRS can monitor cognitive load in real-time in such paradigms.

A key finding of the analysis is the identification of trends related to the connection between the type of disorder, intervention, and reported neurophysiological outcomes. Most studies focusing on MDD used rTMS or antidepressant medication and reported positive associations between hemodynamic changes measured by fNIRS and clinical improvement [37]. In schizophrenia, although methodologies varied, the general trend was similar: improvement in symptoms was associated with positive changes in hemodynamic response. An increase in activation in frontal areas was often observed with treatment, suggesting partial remediation of the initial functional deficit [18,60,72,84]

In contrast, in anxiety disorders, BD, or substance dependence, the application of fNIRS was much more limited in terms of the number of studies, and the results were less consistent.

Clinically, the consistent observation that changes in fNIRS-derived prefrontal activation often parallel symptom improvement, especially in patients receiving rTMS or antidepressants for MDD, supports the potential use of fNIRS as an objective adjunct for tracking treatment response. From this clinical perspective, the observed associations between increased DLPFC activation (as detected by fNIRS) and symptomatic improvement suggest a new avenue for real-time, biological monitoring of psychiatric interventions. If validated in routine care, this could supplement subjective patient self-report and clinical interviews and offer an additional layer of objectivity when evaluating treatment efficacy, particularly in patients who have difficulty in accurately assessing their emotional state, or in situations where placebo effects may confound symptom tracking. The possibility to “see” brain changes in parallel with symptoms improvement could also enhance patient engagement and adherence, as well as understanding and acceptance when treatment adjustments are needed.

However, a main concern remains the fact that more than half of the studies fail to report the direction of fNIRS activation (e.g., increased vs. decreased), and among those that did, both patterns are variably associated with improvement. This heterogeneity in outcome interpretation impedes the development of clear fNIRS-based biomarkers of treatment response.

Overall, while fNIRS holds promise as a monitoring tool in psychiatric research, the current body of evidence reveals critical gaps in methodological rigor and outcome integration. Frequently reported methodological limitations, such as small sample sizes, the absence of control groups, and constraints related to fNIRS instrumentation, indicate a continuing need to strengthen the experimental design. In contrast, the majority of studies, including explicit statements regarding conflicts of interest, reflect a satisfactory level of ethical transparency in the studies reviewed.

## 5. Conclusions Future Directions

This scoping review highlights both the promise and current limitations of fNIRS as a tool for monitoring treatment response in psychiatric disorders. The reviewed studies demonstrate a clear growth in the field, particularly over the last five years. Most studies employed experimental designs, which is a consistent step for establishing a new evidence-based tool in treatment monitoring. However, key limitations persist. A lack of standardization in fNIRS preprocessing and signal analysis pipelines, inconsistencies in reporting activation patterns, and limited integration with clinical outcome data restrict the ability to assess fNIRS as a reliable treatment-monitoring modality. Given the methodological limitations identified and the promising potential of fNIRS in monitoring therapeutic response in psychiatry, future directions should include strengthening clinical validity through rigorous multicenter studies, as well as harmonization in study design and reporting. To do this, integration of multimodal and computational approaches for individualized response prediction and collaborative efforts (e.g., on normative datasets and open-access repositories) might show big potential in supporting generalizability, replication, and meta-analytic efforts.

## Figures and Tables

**Table 1:** Study descriptives.

Variable	Stats/Values	Frequencies (% of Valid)	Graph
Study Design	1. Descriptive/Exploratory 2. Observational 3. Experimental-RCT 4. Non-RCT 5. Pilot	3 (6.4%) 10 (21.3%) 15 (31.9%) 11 (23.4%) 8 (17.0%)	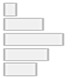
Sample Size	1. 1 2. 2–20 3. 21–50 4. 51–100 5. >100	2 (4.2%) 9 (19.1%) 25 (53.2%) 7 (14.9%) 4 (8.5%)	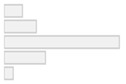
Population Characteristics (adults/children)	1. Adults 2. Children	44 (93.6%) 3 (6.4%)	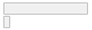
Gender	1. Mixed 2. Female only 3. Male only	40 (85.1%) 2 (4.3%) 5 (10.6%)	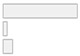
Age Category	1. Children (≤12) 2. Adolescents (13–17) 3. Adults (18–64) 4. Older Adults (≥65) 5. Mixed 6. Not reported	2 (4.3%) 2 (4.3%) 21 (44.7%) 8 (17.0%) 12 (25.5%) 2 (4.3%)	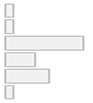
Psychiatric Disorder	1. Major depressive disorder (MDD) 2. Bipolar disorder (BP) 3. Schizophrenia 4. Anxiety disorders 5. Substance use disorders (SUD) 6. Cognitive impairment 7. Other disorders	14 (29.8%) 3 (6.4%) 5 (10.6%) 8 (17.0%) 4 (8.5%) 7 (14.9%) 6 (12.8%)	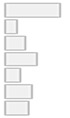
Methodology	1. Quantitative 2. Qualitative 3. Mixed methods	23 (48.9%) 1 (2.1%) 23 (48.9%)	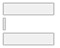
Intervention/ Treatment Type	1. rTMS/iTBS 2. tDCS 3. Medication 4. Psychotherapy 5. VR 6. Other 7. Combined	11 (23.4%) 5 (10.6%) 9 (19.1%) 4 (8.5%) 1 (2.1%) 13 (27.7%) 4 (8.5%)	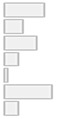
Treatment Duration	1. 1 session 2. >1 session 3. Not reported	7 (14.9%) 38 (80.9%) 2 (4.3%)	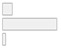
fNIRS protocol	1. Task-based 2. Resting-state 3. Mixed	30 (63.8%) 14 (29.8%) 3 (6.4%)	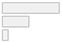
Number of fNIRS Channels	1. ≤8 2. 9–16 3. 17–32 4. >32	8 (17%) 7 (14.9%) 8 (17%) 24 (51.1%)	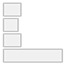
Workload/task	1. VFT 2. Cognitive 3. Emotional/recall 4. VR task 5. Rest 6. Motor 7. Other 8. Combined	14 (29.8%) 6 (12.8%) 5 (10.6%) 2 (4.3%) 11 (23.4%) 1 (2.1%) 1 (2.1%) 7 (14.9%)	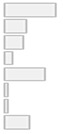
Measurement of Brain Areas/Regions of interest	1. PFC 2. DLPFC 3. OFC 4. Temporal + STG 5. Parietal 6. Multiple 7. Not specified	11 (23.4%) 26 (55.3%) 0 (0.0%) 5 (10.6%) 0 (0.0%) 1 (2.1%) 4 (8.5%)	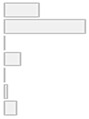
Timepoints Measured	1. Pre/post 2. Longitudinal/multiple 3. Baseline only 4. Single session 5. Other	21 (44.7%) 18 (38.3%) 0 (0.0%) 1 (2.1%) 7 (14.9%)	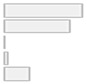
Key fNIRS Findings	1. Increased activation 2. Decreased activation 3. No significant change 4. Mixed 5. Not reported	25 (53.2%) 12 (25.5%) 0 (0.0%) 8 (17.0%) 2 (4.3%)	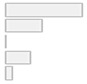
Clinical Outcomes	1. Reported with effect 2. No change 3. Not reported	41 (87.2%) 3 (6.4%) 3 (6.4%)	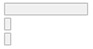
fNIRS—Clinical Link	1. Yes 2. No 3. Not reported	39 (83.0%) 6 (12.8%) 2 (4.3%)	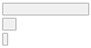
Presentation Software	1. Standard 2. Custom/programmed 3. None 4. Not reported	1 (2.1%) 12 (25.5%) 7 (14.9%) 27 (57.4%)	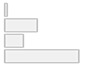
fNIRS Data Analysis Software	1. Toolboxes (e.g., HOMER2) 2. General (MATLAB) 3. Manufacturer software 4. Not specified/Other	13 (27.7%) 8 (17.0%) 19 (40.4%) 7 (14.9%)	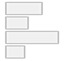
Artefact Removal Procedure	1. Yes (described) 2. No 3. Not reported	35 (74.5%) 3 (6.4%) 9 (19.1%)	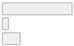
fNIRS Equipment	1. Commercial systems 2. Portable/Wearable 3. Custom-Built 4. Not specified	39 (83.0%) 4 (8.5%) 2 (4.3%) 2 (4.3%)	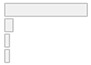
Additional Equipment	1. Neuroimaging (EEG 2. Other types 3. None 4. Multiple 5. Not reported	1 (2.1%) 33 (70.2%) 8 (17.0%) 5 (10.6%) 0 (0.0%)	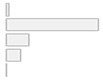

**Table 2:** Limitations reported across studies.

Limitation	Count	Percent
Small Sample	32	45.07
No Control	12	16.90
Follow Up	10	14.08
Technical	10	14.08
Generalizability	6	8.45
Blinding	1	1.41

## Data Availability

Data are contained within this article. A list of included papers from the scoping review can be found in Appendix A Table A2.

## Abbreviations

The following abbreviations are used in this manuscript:

BD	Bipolar Disorder
CCO	Cytochrome-c-oxidase
CSF	Cerebrospinal Fluid
DLPFC	Dorsolateral Prefrontal Cortex
EEG	Electroencephalography
HbO	Oxyhemoglobin
HbD	Hemoglobin Difference
HbR	Deoxyhemoglobin
HbT	Total Hemoglobin
MDD	Major Depressive Disorder
MeSH	Medical Subject Headings
OFC	Orbitofrontal Cortex
PCA	Principal Component Analysis
PET	Positron Emission Tomography
PFC	Prefrontal Cortex
RCT	Randomized Controlled Trial
STG	Superior Temporal Gyrus
SUD	Substance Use Disorder
VR	Virtual Reality
WHO	World Health Organization
fMRI	Functional Magnetic Resonance Imaging
fNIRS	Functional Near-Infrared Spectroscopy
iTBS	Intermittent Theta Burst Stimulation
non-RCT	Non-Randomized Controlled Trial
qEEG	Quantitative Electroencephalography
rCBV	Regional Cerebral Blood Volume
rTMS	Repetitive Transcranial Magnetic Stimulation

## Appendix A

**Table A1:** Keywords and syntax used for each database.

Database	Keywords
Pubmed	(“Spectroscopy, Near-Infrared” [MeSH] OR “Spectroscopy, Near-Infrared” [tiab] OR “functional near infrared spectroscopy” [tiab] OR “fNIRS” [tiab] OR “near-infrared spectroscopy” [tiab]) AND (“Mental Disorders” [MeSH] OR “Persons with Psychiatric Disorders” [MeSH] OR “Mental Disorder” [tiab] OR “Persons with Psychiatric Disorder *” [tiab] OR depression [tiab] OR schizophrenia [tiab] OR anxiety [tiab] OR bipolar [tiab] OR “mood disorder *” [tiab]) AND (“Treatment Outcome” [MeSH] OR “Therapy Monitoring” [MeSH] OR “Treatment Outcome” [tiab] OR “treatment response” [tiab] OR “treatment monitoring” [tiab] OR intervention * [tiab] OR therapy [tiab] OR pharmacotherapy [tiab] OR psychotherapy [tiab] OR neurostimulation [tiab] OR TMS [tiab] OR CBT [tiab] OR TDCS [tiab] OR neurofeedback [tiab])
Web of Science (Abstract)	(“Near-Infrared Spectroscopy” OR “functional near infrared spectroscopy” OR “fNIRS” OR “near-infrared spectroscopy”) AND (“Mental Disorder *”OR “Persons with Psychiatric Disorder *” OR depression OR schizophrenia OR anxiety OR bipolar OR “mood disorder *”) AND (“treatment response” OR “treatment monitoring” OR “intervention *” OR “therapy” OR “pharmacotherapy” OR “psychotherapy” OR “neurostimulation” OR “TMS” OR “CBT” [tiab] OR “TDCS” OR “neurofeedback”)
SCOPUS (Title and Abstract)	TITLE-ABS (“Near-Infrared Spectroscopy” OR “functional near infrared spectroscopy” OR “fNIRS” OR “near-infrared spectroscopy”) AND TITLE-ABS (“Mental Disorder *” OR “Persons with Psychiatric Disorder *” OR depression OR schizophrenia OR anxiety OR bipolar OR “mood disorder *”) AND TITLE-ABS (“Treatment Outcome” OR “treatment response” OR “treatment monitoring” OR intervention * OR therapy OR pharmacotherapy OR psychotherapy OR neurostimulation OR TMS OR CBT OR TDCS OR neurofeedback)
Embase	(mh AND (‘spectroscopy, near-infrared’/exp OR ‘spectroscopy, near-infrared’) OR ‘functional near infrared spectroscopy’/exp OR ‘functional near infrared spectroscopy’ OR ‘fnirs’/exp OR fnirs OR ‘near-infrared spectroscopy’/exp OR ‘near-infrared spectroscopy’) AND ((mh AND (‘mental disorders’/exp OR ‘mental disorders’) OR mh) AND (‘persons with psychiatric disorders’/exp OR ‘persons with psychiatric disorders’) OR ‘mental disorder *’ OR ‘depression’/exp OR depression OR ‘schizophrenia’/exp OR schizophrenia OR ‘anxiety’/exp OR anxiety OR bipolar OR ‘mood disorder *’) AND ((mh AND (‘treatment outcome’/exp OR ‘treatment outcome’) OR mh) AND (‘therapy monitoring’/exp OR ‘therapy monitoring’) OR ‘treatment response’/exp OR ‘treatment response’ OR ‘treatment monitoring’/exp OR ‘treatment monitoring’ OR intervention * OR ‘therapy’/exp OR therapy OR ‘pharmacot herapy’/exp OR pharmacotherapy OR ‘psychotherapy’/exp OR psychotherapy OR ‘neurostimulation’/exp OR neurostimulation OR ‘tms’/exp OR tms OR cbt OR tdcs OR ‘neurofeedback’/exp OR neurofeedback)

* The asterisk (*) is used as a truncation symbol in the search syntax to capture word variations.

**Table A2:** Summary of fNIRS studies.

Author (Year)	Sample	Diagnostic	Treatment Type	Task	Regions of Interest	Equipment	Measurement	Key Findings	Motion Correction
Wong et al. (2021) [42]	70	MDD	Combined	rest	DLPFC	NIRSport (NIRx Medical Technologies LLC)	18	Increased connectivity (resting-state FC) in DLPFC after acupuncture + antidepressant vs. antidepressant alone	Yes
Ho et al. (2025) [43]	64	MDD	Medication	Combined	PSFC, DLPFC, STG, VLPFC, MPFC	ETG-4000 (Hitachi)	52	Increased activation (task-related hemoglobin response in dlPFC) associated with treatment responders.	No
Chou (2023) [37]	26	MDD	rTMS/iBTS	VFT	FT	ETG-4000	52	Increased frontal activation in responders after rTMS, correlated with symptom improvement.	Yes
Yamagata et al. (2019) [44]	11	MDD	Medication	VFT	fronto-temporal cortex	Hitachi ETG-4000	52	Some increased activation post-treatment, but baseline low activation persisted—partial normalization seen (but not total).	No
Blake et al. (2023) [18]	27	Schizophrenia	rTMS/iBTS	rest	DLPFC, IPL	NIRScout system	26	In schizophrenia: initial increase then decrease in IPL activation; healthy controls had immediate decrease. Connectivity abnormality detected (DLPFC → IPL), showing 0mixed pattern.	No
Huang et al. (2025) [34]	272	SUD	Medication	VFT	PFC, right and left temporal lobe, parietal lobe	Hitachi ETG-4000	52	Alcohol-dependent patients showed significantly lower activation in frontal and bilateral temporal lobes compared to healthy controls (pre-treatment baseline data).	Yes
Barrett et al. (2025) [78]	29	BD	Other	Combined	PFC	NIRSIT (OBELAB Inc., Soterix Medical)	48	Decreased prefrontal connectivity (reduction in network correlations) observed post-TILS, despite cognitive improvements.	No
Zhao et al. (2024) [52]	38	Cognitive Impairment	rTMS/iBTS	VFT	DLPFC	NirScan (Danyang Huichuang Medical Equipment Co.)	48	Decreased prefrontal activation (specifically right DLPFC) during verbal fluency task after rTMS.	Yes
Masuda et al. (2017) [36]	147	MDD	Medication	VFT	DLPFC, TC, OFC	ETG-7100	47	Before treatment: hypoactivation in frontotemporal cortex; responders showed slightly greater activation improvement compared to non-responders.	No
Campos et al. (2024) [35]	32	MDD	tDCS	rest	DLPFC	Humon Hex	1	Depression group: increased minimal oxygen saturation (SatO2) and HbO after tDCS; no effect in control group.	No
Wu et al. (2024) [53]	39	BD	Other	rest	PFC (especially DLPFC)	Artinis	24	Increased functional connectivity and left prefrontal activation after γ-tACS.	No
Metzger et al. (2015) [75]	24	Cognitive Impairment	Medication	VFT	PFC, temporal cortex (left and right), Broca’s area, Wernicke’s area, DLPFC, OFC	Hitachi ETG-4000	44	Increase in oxygenated hemoglobin in speech areas; general decrease in prefrontal activation 1elsewhere.	No
Shimizu et al. (2018) [54]	45	Cognitive Impairment	Other	Motor	PFC, DLPFC, mPFC	LABNIRS	45	Increased cerebral blood flow (activation) in PFC during movement music therapy.	No
Feng et al. (2019) [55]	30	MDD	Other	VFT	DLPFC, OFC, VMPFC	FOIRE-3000 (Shimadzu, Kyoto, Japan)	45	Increased HbO in DLPFC, OFC, and VMPFC post-therapy.	Yes
Wu et al. (2022) [56]	1	Anxiety Disorders	tDCS	rest	PFC	NirSmart	45	Decrease in prefrontal connectivity after tDCS.	No
Talamonti et al. (2022) [79]	32	Cognitive Impairment	Other	Cognitive task	PFcd, PFrd, PFrm, PM, M (Brodmann areas 8, 9, 10, 46)	In-house 16 source × 16 detector portable system	256	Significant decrease in cortical activity during cognitive dual-tasks over time.	Yes
Hirano et al. (2017) [32]	138	MDD	Other	VFT	FT	ETG-4000 Optical Topography System	52	Increase in HbO in bilateral frontal cortex during verbal fluency task post-ECT.	No
Deppermann et al. (2016) [17]	83	Anxiety Disorders	Combined	VR	IFG, DLPFC	ETG-4000 Optical Topography System	52	Reduced left IFG activation + increased co-activation between left IFG and contralateral PFC.	No
Ohta et al. (2009) [16]	13	Other Disorders	Other	Emotional/recall	PFC	ETG-100	24	Significant decrease in lateral PFC HbO during trauma recall with eye movements.	No
Shinba et al. (2018) [57]	15	MDD	rTMS/iBTS	rest	PFC	NIRO-300	1	Increase in frontal hemoglobin concentration (fHbC) during TMS linked to symptom improvement.	No
Huhn et al. (2019) [80]	28	SUD	Medication	Cognitive task	DLPFC, PFC	fNIR Devices, LLC (Model 1200)	16	Higher right DLPFC activation during decision-making in cocaine users.	Yes
Huang et al. (2022) [58]	80	MDD	rTMS/iBTS	VFT	DLPFC, VLPFC, FPPFC	BS-3000	37	Increased HbO in bilateral prefrontal regions (FPPC, VLPFC, left DLPFC) post-rTMS.	Yes
Sened et al. (2025) [59]	8	Anxiety Disorders	Psychotherapy	Emotional/recall	IFG	BRITE 24 (Artinis Medical Systems)	24	Inter-brain synchrony increased during therapy (measured by fNIRS hyperscanning).	Yes
Li et al. (2025) [60]	39	Schizophrenia	rTMS/iBTS	Combined	LmPFC, RmPFC, LFEF, RFEF, LDLPFC, RDLPFC, LBroca, RBroca, LFPA, RFPA, LPre & SMA, RPre & SMA	BS-3000	53	Increased activation in PFC; improved small-world network properties (connectivity).	Yes
Yamazaki et al. (2022) [61]	19	MDD	rTMS/iBTS	VFT	PFC	Spectratech OEG-16	16	Post-treatment: increased leftward shift (increased left PFC activation).	No
He et al. (2024) [62]	40	SUD	Other	Emotional/recall	dlPFC, vlPFC, OFC, FPA	NIRScout	20	Increased activation in right dlPFC and vlPFC after HIIT.	No
Lee et al. (2024) [45]	81	Cognitive Impairment	Other	Combined	Prefrontal cortex-right vlPFC, DLPFC	NIRSIT-LITE (OBELAB, Seoul, Republic of Korea)	15	Only predictive features (HbO metrics) reported; no direct pre-post comparison of activation.	Yes
Struckmann et al. (2022) [76]	34	MDD	rTMS/iBTS	rest	dlPFC	NIRO 200X (Hamamatsu, Japan)	2	Connectivity changes: Decreased left dlPFC–insula connectivity after iTBS.	No
Mizumoto et al. (2024) [63]	60	Mixed	Other	Other	orbital PFC	PocketNIRS DUO (Dynasense, Hamamatsu)	2	In depression group: Increased orbital PFC activation; In anxiety group: Decrease linked to better mood.	No
Usami et al. (2014) [64]	10	MDD	Psychotherapy	VFT	frontopolar cortex	2-channel NIRS (Spectratech Inc., Japan)	2	Increased HbO concentration in the frontopolar cortex after 6 weeks of treatment.	No
Taylor et al. (2017) [84]	1	Schizophrenia	tDCS	Cognitive task	Left DLPFC and TPJ	NIRScout (NIRx Medical Technologies, USA)	12	Initial increase in oxygenation during task, followed by decrease post-tDCS.	No
Deppermann et al. (2017) [77]	44	Anxiety Disorders	Combined	Emotional/recall	PFC	ETG-4000 Optical Topography System	52	Increased bilateral PFC activation after verum iTBS.	No
Wigal et al. (2012) [81]	26	Other Disorders	Medication	rest	frontal cortex	Frequency domain NIRS (ISS, Inc., Champaign, IL, USA)	8	Medication induced reorganization of phase synchronization between left and right frontal lobes.	No
Wu et al. (2024) [65]	27	MDD	Other	VFT	PreM, SMA, FEF, Broca	BS-3000	53	Increased hemodynamic response (HbO) and improved functional connectivity after ECT.	Yes
Ruocco et al. (2016) [82]	29	Other Disorders	Psychotherapy	Cognitive task	DLPFC	fNIR Imager 1000r	16	Patients who reduced self-harm showed increased activation in the right DLPFC after DBT.	No
Gong et al. (2022) [66]	45	Other Disorders	Medication	Combined	DLPFC, frontal poles, frontopolar cortex	NirScan6000B (Danyang Huichuang Medical Equipment Co., Ltd.)	48	Increased activation and enhanced connectivity in PFC after drug treatment.	Yes
Huhn et al. (2019) [85]	29	SUD	Medication	Emotional/recall	PFC	fNIR Devices, LLC, Model 1200	16	Increased left lateral PFC activation to drug cues associated with abstinence success.	Yes
O’Donnell et al. (2023) [83]	15	BD	Other	rest	Bilateral anterior PFC (BA 10)	Custom bbNIRS (Ocean Optics QE-Pro)	4	Increased oxidized CCO and HbD (oxygenation) in PFC after TILS.	No
Sutoh et al. (2016) [67]	8	Other Disorders	rTMS/iBTS	Cognitive task	Left and right prefrontal cortex, specifically left DLPFC (Brodmann area 9)	2-channel NIRS (unspecified vendor; forehead mount)	2	Decreased oxygenation in the left DLPFC after rTMS.	No
Tomita et al. (2024) [68]	23	Anxiety Disorders	Other	Combined	rFPA	NIRSport2 (NIRx)	32	Decreased activation in right frontopolar area (rFPA) after tSMS.	Yes
Liu Chen et al. (2024) [69]	28	Other Disorders	tDCS	Cognitive task	dlPFC, FPA, pars triangularis Broca’s area	Preprocessing + GLM (beta values), motion control not specified	19	Increased PFC activation (dlPFC, FPA) after tDCS.	No
Narita et al. (2018) [70]	26	Schizophrenia	tDCS	VFT	Left temporoparietal regions	ETG-4000 (Hitachi Medical Co., Tokyo, Japan)	52	Improvement in psychotic symptoms correlated with baseline HbO but no direct post-treatment brain activation change reported.	Yes
Kim et al. (2024) [71]	45	Anxiety Disorders	Psychotherapy	rest	DLPFC, VLPFC, FPC, OFC	NIRSIT (OBELAB Inc, South Korea)	48	Decreased HbO in right VLPFC and OFC after app treatment.	No
Lee et al. (2021) [46]	74	Anxiety Disorders	VR	VR	FPPFC, OFC, DLPFC, VLPFC	NIRSIT (OBELAB Inc., Seoul, Republic of Korea)	48	Decreased activation in FPPFC and OFC after VR treatment.	No
Gao et al. (2025) [72]	104	Schizophrenia	rTMS/iBTS	rest	DLPFC, bilateral frontal pole, right superior temporal gyrus	NirScan-6000C (Danyang Huichuang Medical Equipment Company)	48	Increased activation in bilateral frontal poles and decreased activation in STG.	Yes
Zhang et al. (2023) [73]	37	Mixed	rTMS/iBTS	VFT	DLPFC, Broca’s areas, Frontal Lobe, FP, SMC, FE, FL	Not specified (likely commercial 53-channel system)	53	Increased functional connectivity between brain regions post-iTBS.	Yes
Liao et al. (2020) [74]	34	Cognitive Impairment	Combined	Combined	PFC	OEG-16 (Spectratech Inc., Japan)	16	Decreased PFC activation after VR training, indicating improved neural efficiency.	No

**Table A3:** The Scoping review checklist.

Key Criteria	Checklist Items	Score One Point for Each Item
1. Study aim, purpose, and research question	1. The rationale/purpose for the scoping review was stated.	1
	2. Appropriate scoping review methodology was used.	1
	3. At least two reviewers conducted the review.	1
	4. The research question(s) was/were used to guide the scope of inquiry (participants, concept, and context included).	1
2. Relevant studies	5. An in-depth literature search was conducted to identify all relevant literature.	1
	6. A comprehensive list of relevant studies that balances breadth with feasibility was identified.	1
3. Study selection	7. The inclusion and exclusion criteria were clearly described and were used to determine eligibility of studies.	1
	8. The study selection involved an iterative process, including searching the literature, refining the search strategy, and reviewing articles for inclusion.	1
	9. At least two reviewers independently reviewed the title and abstracts and reached consensus on studies for inclusion.	1
	10. The study selection process was summarized in a flow chart.	1
4. Charting the data	11. The research team collectively developed a data charting format and determined which variables to extract to answer the research question.	1
	12. The data were charted through sifting and sorting; tables include study details based on full texts.	1
	13. A numerical analysis of the extent and nature of included studies was reported.	1
	14. The quality of papers was assessed.	1
5. Collating, summarizing, and reporting the results	15. Results were presented in a logical descriptive or diagrammatic or tabular format.	1
	16. A narrative account of results was presented.	1
	17. The results were aligned with the review aim, purpose/research question(s).	1
	18. Issues associated with bias were discussed.	N/A
	19. Implications for future research, education, practice, and/or policy were discussed.	1
	20. The conclusion described the current state of the overall literature in relation to the topic.	1
Total		19 points

**Table A4:** Quality of papers assessment.

		Issues	
Studies	No	Yes	Number
Wong et al. (2021) [42]		X	3
Ho et al. (2025) [43]		X	1
Chou (2023) [37]		X	3
Yamagata et al. (2019) [44]		X	2
Blake et al. (2023) [18]		X	5
Huang et al. (2025) [34]		X	1
Barrett et al. (2025) [78]	X		
Zhao et al. (2024) [52]		X	2
Masuda et al. (2017) [36]		X	4
Campos et al. (2024) [35]		X	2
Wu et al. (2024) [53]	X		
Metzger et al. (2015) [75]		X	1
Shimizu et al. (2018) [54]		X	3
Feng et al. (2019) [55]		X	4
Wu et al. (2022) [56]	X		
Talamonti et al. (2022) [79]		X	1
Hirano et al. (2017) [32]		X	2
Deppermann et al. (2016) [17]	X		
Ohta et al. (2009) [16]		X	2
Shinba et al. (2018) [57]		X	3
Huhn et al. (2019) [80]	X		
Huang et al. (2022) [58]	X		
Sened et al. (2025) [59]		X	3
Li et al. (2025) [60]	X		
Yamazaki et al. (2022) [61]		X	2
He et al. (2024) [62]	X		
Lee et al. (2024) [45]		X	3
Struckmann et al. (2022) [76]	X		
Mizumoto et al. (2024) [63]	X		
Usami et al. (2014) [64]		X	3
Taylor et al. (2017) [84]		X	2
Deppermann et al. (2017) [77]		X	2
Wigal et al. (2012) [81]		X	1
Wu et al. (2024) [65]	X		
Ruocco et al. (2016) [82]		X	1
Gong et al. (2022) [66]		X	2
Huhn et al. (2019) [85]	X		
O’Donnell et al. (2023) [83]		X	1
Sutoh et al. (2016) [67]		X	2
Tomita et al. (2024) [68]		X	1
Liu Chen et al. (2024) [69]		X	1
Narita et al. (2018) [70]		X	2
Kim et al. (2024) [71]		X	1
Lee et al. (2021) [46]	X		
Gao et al. (2025) [72]		X	1
Zhang et al. (2023) [73]		X	3
Liao et al. (2020) [74]	X		
